# The Effectiveness of an Integrated Treatment for Functional Speech Sound Disorders—A Randomized Controlled Trial

**DOI:** 10.3390/children8121190

**Published:** 2021-12-16

**Authors:** Denise I. Siemons-Lühring, Harald A. Euler, Philipp Mathmann, Boris Suchan, Katrin Neumann

**Affiliations:** 1 Department of Phoniatrics and Pedaudiology, University Hospital Münster, University of Münster, Malmedyweg 13, 48149 Münster, Germany; euler@uni-kassel.de (H.A.E.); Philipp.Mathmann@ukmuenster.de (P.M.); Katrin.Neumann@uni-muenster.de (K.N.); 2 Department of Clinical Neuropsychology, Ruhr-University of Bochum, Universitätsstraße 150, 44801 Bochum, Germany; boris.suchan@ruhr-uni-bochum.de

**Keywords:** functional, speech sound disorder, phonological, children, treatment, therapy, auditory feedback, randomized controlled trial, integrated, effectiveness

## Abstract

Background: The treatment of functional speech sound disorders (SSDs) in children is often lengthy, ill-defined, and without satisfactory evidence of success; effectiveness studies on SSDs are rare. This randomized controlled trial evaluates the effectiveness of the integrated SSD treatment program PhonoSens, which focuses on integrating phonological and phonetic processing according to the Integrated Psycholinguistic Model of Speech Processing (IPMSP). Methods: Thirty-two German-speaking children aged from 3.5 to 5.5 years (median 4.6) with functional SSD were randomly assigned to a treatment or a wait-list control group with 16 children each. All children in the treatment group and, after an average waiting period of 6 months, 12 children in the control group underwent PhonoSens treatment. Results: The treatment group showed more percent correct consonants (PCC) and a greater reduction in phonological processes after 15 therapy sessions than the wait-list control group, both with large effect sizes (Cohen’s *d* = 0.89 and 1.04). All 28 children treated achieved normal phonological abilities: 21 before entering school and 7 during first grade. The average number of treatment sessions was 28; the average treatment duration was 11.5 months. Conclusion: IPMSP-aligned therapy is effective in the treatment of SSD and is well adaptable for languages other than German.

## 1. Introduction

### 1.1. Developmental Speech Sound Disorders

Developmental speech sound disorders are common in childhood. The ICD-11 (World Health Organization) [1] characterizes them as featuring difficulties in the acquisition, production, and perception of speech that lead to errors of pronunciation, related either to the number or types of speech errors made or the overall quality of speech production. They have long-term negative consequences for a child’s social-emotional, educational, and professional development and are often associated with dyslexia and spelling disorders later in life [2,3,4]. Their prevalence is reported as ranging from 2.3% to 24.6% [5], though these values differ between studies and vary by child age, with most studies reporting a prevalence between 6 and 16% [6,7,8,9]. Developmental speech sound disorders have been reported to account for an estimated 75% of all communication disorders in children [10]. The Dutch Youth Health Service, for example, reported a ratio of 49% articulation problems to 9% developmental language disorders in a population of 5-year-old Dutch children [11].

Speech sound disorders are among the most commonly treated developmental disorders in childhood. The Remedies Report by the largest health insurance company in Germany, for example, reported that 24.1% of boys and 15.2% of girls aged 6 years received speech therapy in 2018 [12].

The American Speech–Language–Hearing Association [13] divides speech sound disorders into two categories: (1) organic disorders with structural motor, neurological, sensory, or perceptual causes; and (2) functional disorders with no known causes. The study presented here focuses on the latter.

Developmental functional speech sound disorders, hereafter referred to simply as SSDs, are heterogeneous and include problems in the motor production of speech sound movements as well as linguistic difficulties in speech production [13]. SSDs are traditionally grouped into articulation disorders and phonological disorders. The former refers to errors in the production of individual speech sounds, such as distortions and substitutions, while the latter refers to predictable, rule-based errors occurring when using speech sounds in a target language, such as fronting, stopping, and final consonant deletion [13,14]. The term “phonological processes” describes regular differences between child and adult speech during language development. Phonological processes imply the systematic simplification of patterns of adult speech by children, for example, by omitting or substituting sounds, and are not a consequence of speech motor deficits. The age-related limits of typical phonological processes are defined as processes that are shown by less than 20% of typically developed children at this age [15]. If these phonological processes are not overcome during typical developmental periods of language acquisition, they are referred to as phonological disorders, which are a type of developmental language disorder. Children with SSD show a higher number of phonological processes than their typically developing peers [16]. However, because it is often difficult to clearly distinguish between articulatory and phonological disorders, the term “speech sound disorder” has been used as an overarching term for speech errors of unknown cause [13].

### 1.2. Evidence for Treatment Effects in SSD

A meta-analysis of five treatment efficacy studies on developmental speech and language delay disorder in children [17] demonstrated positive outcomes with moderate effect sizes supporting the benefit of phonological interventions for expressive phonological disorders in childhood SSD over no intervention. No significant improvements in productive phonology were found for interventions administered by parents based on receptive hearing techniques. However, the generalizability of these findings could be disputed owing to the small sample sizes used and the frequent lack of detail provided about treatment methods, meaning that the replicability of these treatment approaches may be limited. The reported studies refer exclusively to English-speaking children. A further meta-analysis by Nelson, Nygren, Walker, and Panoscha [18] reported treatment-related improvements in articulation and phonology in children aged from 3 to 5 years across multiple therapeutic settings, for example, using interventions such as the Derbyshire language scheme and modeling techniques [19] or a combination of minimal pairs and traditional articulation therapy [20].

A systematic review by Wren, Harding, Goldbart, and Roulstone [21] gave labels to five categories of intervention used in the diverse range of treatment procedures for SSD in preschool children: four approaches that focus on specified directed activities (the auditory-perceptual, cognitive-linguistic, production, and integrated approaches) and the environmental approach. Three studies within the auditory-perceptual [22] and integrated approach categories [20] yielded the highest scored evidence for treatment effectiveness, including from one 8-month follow-up study [19].

Treatment approaches that are based on training the differentiation of minimal pairs (sets of words differing by a single phoneme that is sufficient to indicate a change in meaning) have been used for several decades [23]. In their narrative review, Baker and McLeod [24] identified 134 treatment studies for SSD with 46 different approaches and differing levels of evidence provided, with the minimal pairs approach being the most frequently studied.

The environmental approach [21] uses everyday actions and includes modeling and recasting activities, in which the child’s speech is newly formed (modeling) by the repetition by an adult of the child’s incorrect speech production in a corrected version (recasting). This approach, amongst others, has been studied by Yoder, Camarata, and Gardner et al. [25]. Children with severe phonological and expressive language impairment were presented with grammatical recasts after well-articulated child utterances and speech recasts after poorly articulated ones. This approach improved speech intelligibility and mean length of utterance (MLU) post-treatment, but only for a subgroup of children with low pre-treatment articulation scores. The treatment effects were only small to moderate after eight months of follow-up [25].

Auditory-perceptual treatment involves auditory stimulation and discrimination tasks. Rvachew and colleagues [22] found greater improvements in phonemic perception and articulatory accuracy using this approach when additional training of letter recognition, letter–sound association, and onset-rime matching was included. No benefit was found for stand-alone phonological awareness training.

In the Metaphon approach [26], which is a cognitive-linguistic intervention, specific training of metaphonological awareness is added to the training of minimal pairs. This treatment focuses on enhancing phonological and communicative aspects of language. In the first phase of treatment, the intention is to develop the child’s awareness of the properties of speech sounds and to broaden their interest in the speech sound system. In the second phase, the child is guided by the speech–language therapist (SLP) to recognize their own phonological failures and correct them. Fox-Boyer [15] noted that the Metaphon approach requires that children already have good cognitive and abstraction skills and that it may not be suitable for young children or those with cognitive restrictions. Not all kinds of phonological errors are treatable using this approach, for example, onset processes in which many different initial consonants are replaced by [d] or [h].

In production approaches, oromotor tasks, phonetic modeling, imitation, and drills are used (e.g., [27]). In this variant of the core vocabulary approach, the aim is to acquire phonological production (although this production must not necessarily be correct). Drilling of core vocabulary is used to help the acquisition of consistent phonological output plans. The effectiveness or efficacy of this treatment has been documented by an RCT [6], an experimental trial [28], and four quasi-experimental trials [27]. Fox-Boyer [15] questioned the transferability of the core vocabulary approach to the German language because it does not do justice to the German language structure. She reported single cases treated with a core vocabulary-like concept, which showed improvement only for words of up to three syllables but not for multisyllabic words or words with a prefix, which are common features of German.

Integrated interventions combine two or more of the four specific directed procedures [21]. The accuracy and stability of a child’s articulation are linked to their developing perceptual knowledge about phonemes [22]. This link supports the validity of integrating phoneme perception and production into the treatment of SSD. Integrating these features can be achieved successively, as in the treatment approach of Almost and Rosenbaum [20]. Their RCT of preschool children with SSD demonstrated the effectiveness, in comparison with non-treated children, of an integrated intervention featuring an initial phase teaching minimal pairs followed by a phase of traditional articulation treatment. In a study by Pamplona, Ysunza, and Espinosa [29], children with cleft palate and compensatory articulation disorders received integrated training of speech perception and production and required significantly less treatment time than children who underwent pure production training. One Portuguese study featured integrated training of expressive phonological tasks, phonological awareness, listening, and discrimination activities and demonstrated some benefits for children with SSD [30]. Dodd and Bradford [31] concluded from a study, in which they sequentially applied three different treatment approaches to children with phonological disorders, that some children may need the selection and sequencing of a range of different treatment approaches since different parts of a child’s phonological and phonetic system may respond to different types of approaches that target different aspects of speech production.

The background described above formed the basis for our assumption, in designing the PhonoSens treatment, that an integrated treatment approach for SSD that combines the training of categorical phoneme perception, phoneme production, auditory self-monitoring, and orosensory facilitation may be superior to approaches that cover only one aspect of speech sound acquisition. These aspects are explained in more detail below. These findings also informed our decision to design the treatment method examined in our study so as to incorporate the auditory-perceptual and integrated approaches—those that yielded the strongest evidence of effectiveness in the systematic review by Wren et al. [21]—by integrating phonological and phonetic processing skills, including categorical phonological awareness and intensive auditory–somatosensory self-observation training.

### 1.3. New Treatment Concepts for SSD

Previous classifications of SSD focused heavily on the separation of phonological and phonetic aspects [32]. One example of this is Dodd’s [33] model of differential diagnosis, which classifies SSD into four phonological subgroups: (1) Phonological Disorder, (2) Phonological Delay, (3) Consistent Phonological Disorder, and (4) Inconsistent Phonological Disorder. It also differentiates between phonological disorders, Phonetic Articulation Disorders, and Developmental Apraxia of Speech. The separation of phonological and phonetic aspects has also been reflected in the conceptualization of the SSD treatment as reported by meta-analyses and systematic reviews [17,21]: only 3 out of 26 studies evaluated treatment approaches that integrated phonological and phonetic aspects. Approaches that integrated phonetic production and auditory-perceptual exercises provided the best results [20,21,29].

The phonological view of the origin and treatment of SSDs results mainly from analysis of the outcome, i.e., a child’s speech production. Classifications such as that of Dodd [33] aim to do justice to the presumed causes of SSD but might not take into account the complex cooperation between the different levels of speech processing and production. One further difficulty is that mispronunciation may have several conceivable causes. For example, if a child consistently says /t/ and /d/ instead of /k/ and /g/, this may have phonological causes (the child may not have developed meaningful distinctive phonological categories for /k/, /t/, /g/, and /d/). A plausible alternative explanation would be that the child may have difficulty in motor programming, planning, or execution and therefore simplifies their motor movement pattern by using only two articulatory targets instead of four. A combination of both causes is also possible. In rare cases, children show a gagging reaction when trying to form a velar sound. This may be due to a hyperactive gag reflex [34]; however, it is also possible that the child is trying to form the velar sound at relatively deep posterior trigger regions of the oropharynx and is thereby triggering the gag reflex. There are controversial views on this in the literature [35].

Namasivyam and colleagues [36] support the idea that phonetics and phonology are intertwined, and they describe this in their Articulatory Phonology model (AP model). They hypothesize that there is synergy between the development and refinement of phonological contrasts and speech motor control. In this context, the development of speech sounds is closely associated with the development of tongue–jaw mobility. They suggest that children with SSD are at the lower end of the speech motor continuum, a position that is similar to the assumed mechanism around stuttering [37,38]. The different manifestations of SSD would reflect the different coping strategies that children have developed in order to deal with their speech motor limitations.

In everyday SSD therapy, phonological methods are often combined into a hybrid treatment with classical articulation therapy. This has been asserted by a study [39] in which semi-structured, in-depth individual interviews with 11 Australian SLPs were analyzed. The therapists reported using SSD treatments combining components of phonological-perceptual therapy with classical articulation approaches and tailoring them to the needs of individual clients. This finding suggests that there is a need in clinical practice for SSD treatment methods that combine phonological and phonetic components in an effective, structured manner [39].

Computational experiments [40,41] have indicated that the development of speech sound production is a complex process in which integrated feedforward and feedback subsystems are active. Tourville and Guenther [42] showed the interconnectedness of the phonological and phonetic levels being governed by different feedback mechanisms in a neural network model for the acquisition of speech motor skills and speech production (Directions Into Velocities of Articulators, or DIVA). The implication for speech sound acquisition is that children must not only learn language-specific meaningful acoustic discrimination categories (phonological targets) but also how to translate the phonological targets into adequate articulatory movements (phonetic learning). The child needs control mechanisms to detect and correct errors for this process, as well as constant feedback on the position and state of the articulators.

Terband [32] introduced the Integrated Psycholinguistic Model of Language Processing (IPMSP), which combines cognitive and sensorimotor functions with a hierarchical control system consistent with the view outlined above. The IPMSP is an adaptation of models of speech processing from Levelt [43] and Guenther [40,41]. In the IPMSP speech production model, a chain of four subsequent processing levels is assumed to be active, presumably controlled by three monitoring levels. In the beginning, lexemes (abstract units of the mental lexicon that carry a conceptual meaning) are retrieved from the lexicon. Lexemes form the input for the phonological encoding level in which the sensorimotor targets are selected, sequenced, and temporarily stored. This requires clear, meaningful phoneme categories (the phoneme is the smallest unit of sound within a spoken language with a meaning-differentiating function), determination of the appropriate phonemes in the correct order, and the ability to store this information in a short-term memory buffer. At the next level of motor planning, articulatory movements are selected and sequenced, with the requirements of coarticulation taken into account (coarticulation means that a single speech sound is influenced by and becomes more similar to a preceding or subsequent speech sound) [44]. It is, for example, easier to correctly produce /t/ after /n/ in the syllable /unt/ than after /k/ in the syllable /akt/. Coarticulation is successful when smooth transitions between phonemes are present. At the level of motor programming, motor plans are translated into muscle-specific motor programs, while sensory information and metalinguistic requirements, such as prosody and speech rate, are integrated. At this stage, external circumstances are also taken into account, such as the adjustment of the speech volume to the ambient sounds. Ultimately, during motor execution, neural signals are sent to the peripheral systems, and the appropriate movements are performed. The accuracy of coordinated muscle activity is reflected in diadochokinesis (the ability to perform antagonistic movements, for example of the articulators, in quick succession) and the maximum repetition rate of syllables.

Various internal and external control systems are active in monitoring this ongoing chain of speech production processes. The internal feedback loop monitors motor planning and enables preverbal error repair by checking the phonemic integrity of the speech plan, thus allowing detection and correction of encoding errors just before articulation. The external feedback loop monitors both the fast sensorimotor and slower auditory processes while providing constantly updated information about the status and position of the articulatory organs to the level of motor programming. Likewise, the level of phonological encoding and the higher linguistic levels continuously receives information via the external feedback system, for example, for error correction during or after speech production. Impaired internal self-monitoring leads to poor preverbal monitoring characterized, for example, by the absence of uniqueness point effects (the phoneme position in words from which the word differs from all other words) when judging which of two phonemes is present in a word [45]. Impaired articulatory adaptation may be a sign of impeded external auditory feedback.

Although this complex system of speech processing is very robust in adults, a disruption or weakness at even a single level during childhood development of speech sound processing affects all subsequent systems and impedes the child’s learning. The treatment of SSD should, therefore, not focus exclusively on one aspect of the speech sound production complex, nor should it be guided solely by the child’s performance. Treatment should focus on the first deficit in the hierarchical structure of the speech production chain and ensure that subsequent levels can be addressed gradually [32]. The various internal and external control mechanisms that govern these processes should also be integrated into treatment. Auditory self-monitoring skills have proven to be important for the generalization of target phonemes in spontaneous speech, which is the ultimate goal of treatment [11,46,47].

In accordance with this concept, we have developed the PhonoSens integrated treatment program for SSD in children, which gradually targets different levels of speech sound processing, strengthening sensorimotor feedback, and improving internal feedback control as well as external auditory feedback control.

Some methods use visual symbols as therapeutic aids to help children identify specific sound characteristics. In the Metaphon approach [26], pictures symbolize individual phonological classes. Rvachew et al. [22] used letters to refer to individual phonemes. The PhonoSens treatment uses non-speech, sound-symbol pictograms (SSP cards), such as a picture of a dripping water tap for /t/, to refer to individual phonemes, similar to the letters used in the above-mentioned Rvachew study. Each individual phoneme is represented by an SSP card, which emphasizes the unique categorical assignment of each phoneme. The SSP cards are used in therapy to support perceptual and production exercises and to help a child to categorize, identify, and distinguish target phonemes from error phonemes.

Another important factor in SSD treatment is the sequencing of target phonemes. This sequencing can have a significant impact on the spontaneous improvement of non-treated phonemes, potentially reducing the total number of treatment sessions required. It has traditionally been assumed that the sequence of target phonemes should follow phonological development. However, this notion has been challenged by a recent shift of attention towards structural linguistic complexity. Here, Gierut and Champion showed that treatment of word-initial three-element clusters in children with SSD led to widespread generalization to untreated two-element clusters and singletons, including affricates, suggesting a segmental-syllabic interface based on the notion of linguistic complexity [48]. However, despite good supporting evidence [49], the complexity approach is not yet common in everyday practice.

### 1.4. Efficacy vs. Effectiveness Studies and the Need for Precise Description of Interventions

Treatment effects can be reported either by efficacy or effectiveness studies [50]. Efficacy studies are conducted under well-controlled research conditions and report whether a treatment can work. The question of whether or not a treatment *does* work in daily practice is answered by effectiveness studies [51]. Most treatment studies into speech–language disorders are of the efficacy type. However, effectiveness studies are the most relevant to healthcare practice, and—as urged by several authors [17,52]—there is a need to study the effectiveness of interventions for speech–language disorders using well-described and replicable treatment components and specifications. This is also true for SSD, the treatment of which is often lengthy, procedurally ill-defined, and lacks satisfactory evidence of success. The study reported here is an effectiveness study with well-manualized treatment steps.

### 1.5. Aim of the Study

There is a need for studies evaluating integrated treatment approaches for SSD based on current psycholinguistic models of language processing. Randomized control trials (RCTs) on SSD are rare in general and completely lacking for German-speaking children. This RCT aims to examine the effectiveness of PhonoSens, a newly developed integrated treatment for developmental SSD, in German-speaking children in the daily practice of speech–language therapists. We hypothesized that the PhonoSens treatment would be more effective in increasing the percent correct consonants (PCC) and reducing the number of phonological processes after a period of 15 treatment sessions, compared with a wait-list control group. We also sought to identify the average number of treatment sessions required to reach the success criterion, i.e., the generalization of all target phonemes in spontaneous speech.

## 2. Materials and Methods

### 2.1. Treatment Procedure of PhonoSens

The concept of this treatment approach is in accordance with the speech processing chain described in the IPMSP as depicted in Table 1. The strengthening and development of internal and external feedback systems are integrated from the beginning of treatment onwards. The therapist encourages the child to produce the target phoneme in turn-taking activities from the very beginning. Feedback from the therapist enables the child to compare, train, and adapt their own feedback mechanisms for both preverbal monitoring and external auditory feedback.

A protocol guides the therapist through the treatment steps, and deficits identified are improved sequentially. Perceptual and production skills are trained using turn-taking activities, as are the corresponding monitoring systems. The onset of generalization of a target phoneme is marked by the correct production of the previously incorrectly produced phoneme in untrained words and is believed to occur when correct realizations of the target phoneme appear in three different untrained words during brief conversational episodes between and within exercises. The number of times that three correct realizations of a previously incorrectly produced target phoneme occur within different words in spontaneous speech provides a standardized (though arbitrarily determined) target to ensure therapeutic success in brief dialogues during therapy. Once generalization has begun, it is expected to lead to further generalization of the target phoneme in different articulatory contexts [53]. In this case, the current target phoneme can be abandoned immediately, and treatment of the next target phoneme can begin.

PhonoSens treatment focuses on single target phonemes. Once the first target phoneme is pronounced correctly at the word level, a second target phoneme is included in the treatment. Thus, two target phonemes can be treated simultaneously in different treatment steps. The structure of PhonoSens allows the inclusion of phonemes that the child can already produce correctly (stimulable) as well as phonemes that the child cannot yet produce correctly (non-stimulable). In this way, all affected phonemes can be treated regardless of the type of deviation.

The selection of target and error phonemes is based on the German speech sound acquisition test PLAKSS (Psycholinguistic analysis of children’s speech sound disorders; in German *Psycholinguistische Analyse kindlicher Aussprachestörungen*) [54]. Stimulable sounds that correspond to the target phonemes are given initial priority because the aim is the child’s early phoneme production. Subsequent target phonemes with a high degree of phonetic similarity to the correctly produced phonemes within the child’s phonemic inventory are selected. Target phonemes that are acoustically distinct from the error phoneme are selected later. The remaining target phonemes are given low priority. This procedure reflects the importance of phoneme production for the development of self-monitoring and the improvement of phonological categorical awareness. Other factors, such as the visibility of a phoneme, only play a role if they can positively support the stimulability of the phoneme or facilitate the acoustic differentiation between error phonemes and target phonemes. PhonoSens neither follows the developmental nor the complexity approach because stimulability is the most important criterion for the selection of target phonemes.

The PhonoSens treatment uses reference cards of non-speech sounds, such as a pictogram of a dripping water tap for [t]. All target and error phonemes are represented by non-speech sound-symbol pictograms (SSP cards). Distorted phonemes are represented by slightly incorrect pictograms, such as a water tap pointing up instead of down.

**Figure 1:**
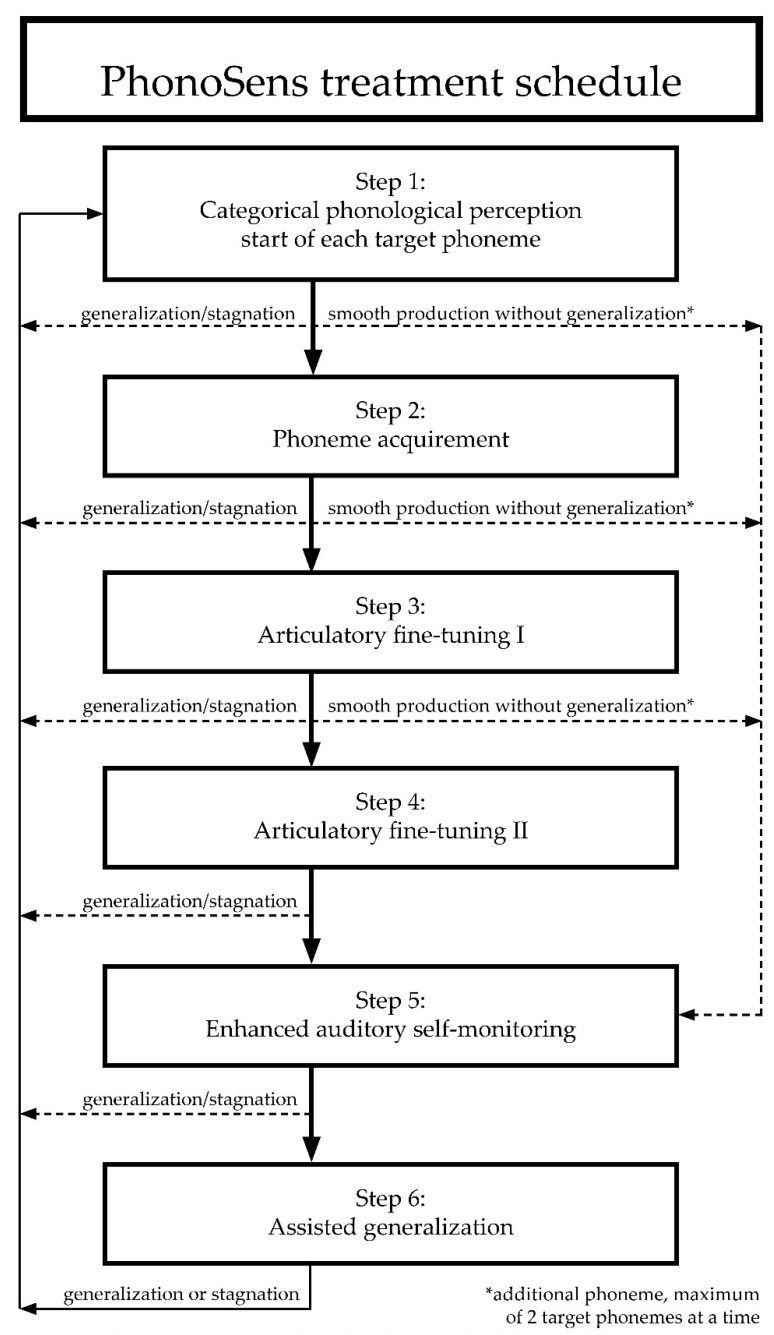
PhonoSens treatment schedule with hierarchical level and instruction for individual adaptation (dashed lines) over the course of a child’s treatment.

The structure of the PhonoSens treatment allows for an individualized pathway for each child and for each target phoneme. At the end of each step, predefined criteria lead to the next treatment step. Treatment of each target phoneme begins with Step 1 (Figure 1), with subsequent steps being based on the child’s individual progress.

#### 2.1.1. Steps of the PhonoSens Treatment

##### Phonological Encoding—Treatment Step 1: Categorical Phonological Perception

Children with SSD show abnormal categorical speech sound perception skills [55,56]. Categorical perception describes the formation of categories for variables that move along a continuum. For different phonemes, different boundaries are conceivable. For example, for bilabial explosive sounds, a binary categorization depending on the time of sonication could yield the categories /b/ and /p/. However, a tripartite categorization would also be possible if sonication occurs later. In this case, /p/ would be perceived as aspirated /p^h^/. At the level of phonological encoding, it is necessary to teach a child clearly identified phonological categories for the target phoneme and the competing error phonemes(s) they have produced (substitute phonemes or distorted versions of the target phoneme). If a child says /tu:/ instead of /ku:/ (in German “cow”), the error phoneme /t/ must be perceptually and productively separated from the target phoneme /k/. It is not sufficient to simply identify the error /tu:/ instead of /ku:/ in another speaker; the child must master these phonological categories in her or his own speech production. The same procedure applies to a distortion or an omission of the target phoneme. For example, if a child produces a nasalized version of the target phoneme /k/, a distinction must be made between the distorted and the correct version of the target phoneme /k/. If a child omits /k/, categorization will focus on the contrast presence or absence of the target phoneme, as in the minimal pair /kauf/ (in German “buy”) or /auf/ (in German “on”). In addition, children must learn to recognize the location of the phoneme in the word. They do not necessarily have to tell the therapist that the phoneme occurs initially, medially, or finally; it is sufficient if they try to produce the target phoneme at the correct position in the word.

##### Motor Planning—Treatment Step 2: Phoneme Acquirement

If a child can categorically differentiate the target phoneme from the error phoneme(s) but cannot correctly produce the target phoneme, step 2 (phoneme acquirement) should be performed. This step corresponds to the motor planning level of the IPMSP. The correct production of the target phoneme is practiced in many small steps beginning from isolated production, progressing through a syllable transition level up to word and sentence levels. For some children, this is accomplished within a few minutes; others need intensive training for every single level.

##### Motor Programming—Treatment Step 3: Articulatory Fine-Tuning I

If a child requires several trials or if they stop before producing a word in order to ensure correct completion of the target phoneme, smooth production of the target phoneme has not yet been mastered. In the first step of articulatory fine-tuning, training of additional words and sentences allows the child to improve the coarticulatory transitions of the target phoneme in a rich phoneme context.

##### Motor Execution—Treatment Step 4: Articulatory Fine-Tuning II

Difficulties at the level of motor execution manifest themselves in coordination deficits, as observed in diadochokinesis tasks or measured in maximum syllable repetition rate. This level is trained by having the child speak the same word three times rapidly in one breath. Multiple repetitions of a word facilitate fine-tuning, as each change can be tested directly and further refined as needed.

##### Treatment Step 5: Enhanced Auditory Self-Monitoring

If internal self-monitoring and external auditory self-monitoring are not sufficient for the generalization of a target phoneme in spontaneous speech, step 5 (enhanced auditory self-monitoring) is necessary. This step involves exercises in identifying target or error phonemes before, during, and after words are spoken.

##### Treatment Step 6: Assisted Generalization

In this last part of the training, the generalization of the correctly produced target phoneme in everyday speech is promoted by targeted rewarding.

##### Sensorimotor Feedback—Thermal Tactile Oral Stimulation (TTOS)

Sensorimotor feedback provides information about the state and position of the articulators. This information is essential for motor programming. In order to support sensorimotor feedback, orosensory perception is stimulated with an ice stick (TTOS) for a few minutes at the beginning of each session. Evidence for this procedure comes from dysphagia research, where TTOS has been shown to facilitate both the oral and pharyngeal phases of deglutition and to produce a significant bilateral increase in cortical activation [57]. This activation occurs predominantly in left-hemispheric somatosensory cortical areas during the voluntary oral phase of swallowing. We, therefore, hypothesize that increased activation of these areas may also support somatosensory feedback during speech sound production.

Other studies have provided evidence for a close relationship between mechanically induced somatosensory change, speech perception, and speech motor adaptation. Ito and colleagues [58], for example, reported a surprising change in the perception of speech sounds affected by subjects listening to the words “head” and “had” while a robotic device stretched the skin on their face in a specific deformation pattern corresponding to the appurtenant speech sound. This change in perception was only observed during the time window of skin stretching. Similar results were obtained in another study using mechanical pertubations of facial movements (Nasir & Ostry, 2009) [59]. A robotic device was used to displace the jaws of adults whilst repeating the words “had”, “sad”, “mad”, and “bad” several times. After the subjects had successfully adapted their articulation to the jaw displacement, it was found that their categorical perception of the words “had” and “head” had changed. Subjects who did not adjust their articulation showed no changes in speech perception. We, therefore, assume that the cold stimulus in TTOS may improve somatosensory perception and thus support speech motor adaptation. Motor learning is, of course, complex and includes other processes besides somatosensory and auditory feedback that need to be improved by targeted training.

##### Treatment Dose and Division

In a review of 206 phonological intervention studies, Sugden et al. [60] found that over 77% of studies reported no data on the dose of productive practice provided per session and even that over 94% reported no data on the dose of perception exercises provided per session. On the basis of three phonological intervention studies (multiple oppositions, minimal pairs), Williams [61] recommends a minimum dose of 50 trials per 30 min session for moderate-severe SSD and at least 70 trials per 30 min session for severe SSD. In PhonoSens, therapy is divided approximately in half between perceptual and production training, with roughly 80–120 trials per 45 min session.

### 2.2. Participants

The parents of children, who were between 3.5 and 5.5 years old and attended one of the five participating kindergartens, received a letter of invitation for a child language screening. In total, the parents of 96 children (median 4.5 years, range from 3.5 to 5.3) signed an agreement to have their child tested with the Child Language Screening (*Kindersprachscreening*, KiSS) [62]. The KiSS test includes, among other subtests, an 11-item articulation subtest. Forty-two children failed the articulation part of the screening test and participated in a comprehensive language test shortly thereafter. Detailed examination of speech sounds, performed with the PLAKKS, revealed that 32 children (14 boys, 18 girls, median 4.6 years, range from 3.6 to 5.5 years) had a delay of at least six months in their articulation skills, leading to a diagnosis of SSD. They were randomly assigned to the treatment group (median age 4.6 years, range from 3.6 to 5.5 years) or the wait-list control group (median age 4.6 years, range from 3.8 to 5.1 years). Genders were equally distributed across both groups: nine females and seven males each. The large number of confirmed SSD cases suggests that it was mainly parents who were concerned about their child’s language development who agreed to participate in the study.

An audiometric test by the child’s pediatrician or ENT doctor indicating normal hearing was required, as was confirmation from a pediatrician of the absence of structural speech problems due to speech motor, anatomical, or intraoral sensory difficulties, as well as general developmental delay and neurological disorder. The participants had not received treatment previously for SSD, and they spoke German as their first language. Written informed consent was obtained from all parents prior to recruitment.

### 2.3. Data Collection

Participating children were shown the images used in the PLAKSS test by the therapist and verbally named them. These verbal responses were recorded using an Olympus VN-8500PC digital voice recorder. An independent, specially trained therapist transcribed the tapes phonetically using the International Phonetic Alphabet. The therapist identified error phonemes, sigmatisms, and other distortions.

#### Time Course of Critical Target Testing

The assessments were performed at three time points. The initial testing at T0 was followed by a period in which the 16 children in the treatment group received 15 weekly PhonoSens treatment sessions of 45 min each. This number of treatment sessions allowed for demonstrable success and seemed feasible for most participants without risking a high number of dropouts; it also covered a not-unreasonable period of time for children in the wait-list control group to not receive speech therapy. The children did not receive any additional therapy. After 15 sessions (T1), their articulation was tested again with PLAKSS. Children who still showed a delay of at least 6 months in their articulatory abilities received further treatment. The 16 children in the wait-list control group were also tested with PLAKSS at T1 (end of waiting period). As a result, one child no longer needed treatment (as judged by the therapist), the parents of two children no longer considered treatment necessary, and one child dropped out after seven sessions. The 12 remaining wait-list control children completed their 15 weekly treatment sessions and were retested with PLAKSS (T2 testing). Following this procedure, the children in the wait-list control group received three tests, and the children in the treatment group received two tests.

Treatment frequency was scheduled once a week, as is common in Germany [63]. Because of illnesses and holidays, the actual weekly treatment frequency was 0.7 on average, with a range of 0.5–1.0. The mean duration from T0 to T1 was six months for both the treatment (*SD* = 1.4) and wait-list control groups (*SD* = 1.8), with a range of 4–9 months.

### 2.4. Therapists

Four experienced therapists received a 2-day training session and practiced the PhonoSens treatment procedure (shown in Figure 1) with children for 3 months prior to the study. During this training period, the therapists participated in monthly feedback sessions. After recruitment, participating children were randomly assigned to the therapists.

### 2.5. Outcome Measures, Statistical Analyses

PCC scores (dependent variable) were calculated for all consonants and consonant clusters tested (*n* = 224) for the items of the PLAKSS articulation test. There was no significant difference (*t*-test, *p* = 0.37) in initial PCC scores (T0) between the treatment group (80.9) and the wait-list control group (84.3). Table 2 lists the incorrectly spoken consonants for each child and the number of incorrectly spoken consonants at T0 and T1. The number of phonological processes was determined for all consonants and consonant clusters marked as incorrect in the PLAKSS articulation test. There was no significant difference in the number of phonological processes between the treatment group (mean 44.4, *SD* = 29.0) and the wait-list control group (mean 37.0, *SD* = 24.4) at T0, as calculated by a *t*-test (*p* = 0.51). Table 3 shows the number of phonological processes for each child at T0 and T1. The number of phonological processes was included as a second dependent variable.

One additional outcome measure was the number of sessions required before the success criterion (defined as the occurrence of at least three correct utterances of each target phoneme in the child’s spontaneous speech within a session) was achieved.

The statistical analyses were performed as one-way between-group ANOVA or MANOVA using SPSS (v.20 for Windows, SPSS Inc., IBM, Ehningen, Germany). Effect sizes were calculated as Cohen’s *d*. Ethical approval for this study was obtained from the Ethical Board of Goethe University, Frankfurt/Main, Germany, registration number 313/10.

## 3. Results

### 3.1. Scores for Percentage of Correct Consonants (PCC)

**Figure 2:**
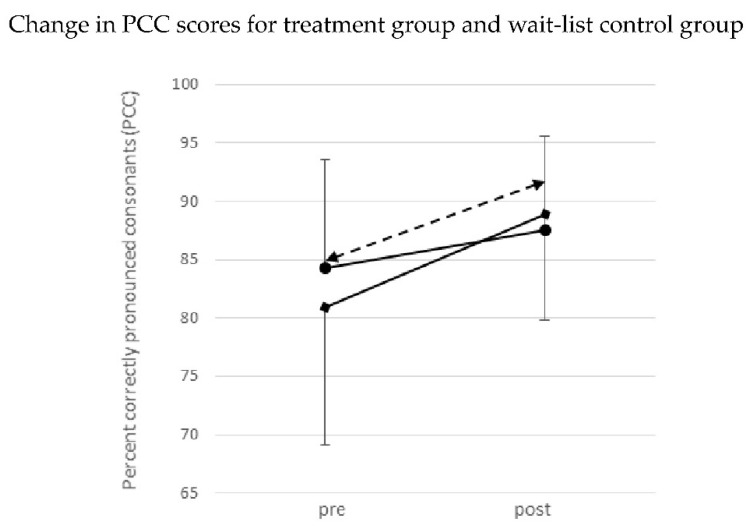
Change in percent correctly pronounced consonants (PCC) from time point To to T1, for the mean of the treatment group and the wait-list control group (solid lines). Change of PCC scores from time point T1 to T2 for the wait-list control group after their treatment phase following the waiting phase is shown as dashed line.

The change in PCC values between T0 and T1 was normalized by a square root transformation. A one-way ANOVA between groups was calculated, with the difference in PCC scores as the dependent variable and group (treatment vs. wait-list control) as the independent variable. The ANOVA found a main effect for group, *F*(1,30) = 7.42, *p* = 0.01, and a partial *η^2^* = 0.20. The treatment of the wait-list control children (*n* = 12) began at T1. From T1 to T2, the mean PCC values of the now-treated children in the wait-list control group increased from 84.9 (*SD* = 7.6, range 74.1–96.9) to 92.7 (*SD* = 5.6, range 77.2–98.7). Compared to the treatment group, the children in the wait-list control group showed comparable progress in PCC values during their treatment phase (T1 to T2). The changes in PCC values over both treatment phases and the waiting phase are shown in Figure 2 (means and *SD*). Details of incorrect consonants are given in Table 2.

**Figure 3:**
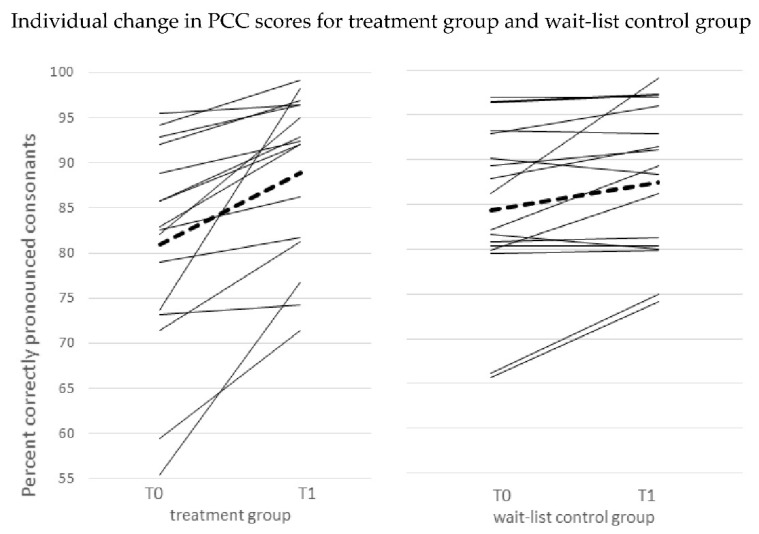
Change in PCC scores for each participant from time point To to T1, for treatment group and wait-list control group; solid lines: individual scores; dashed lines: means.

The individual scores for change in PCC values over time from T0 to T1 (Figure 3) show improvements for all 16 children in the treatment group but only for 11 out of 16 children in the wait-list control group.

### 3.2. Number of Phonological Processes

**Figure 4:**
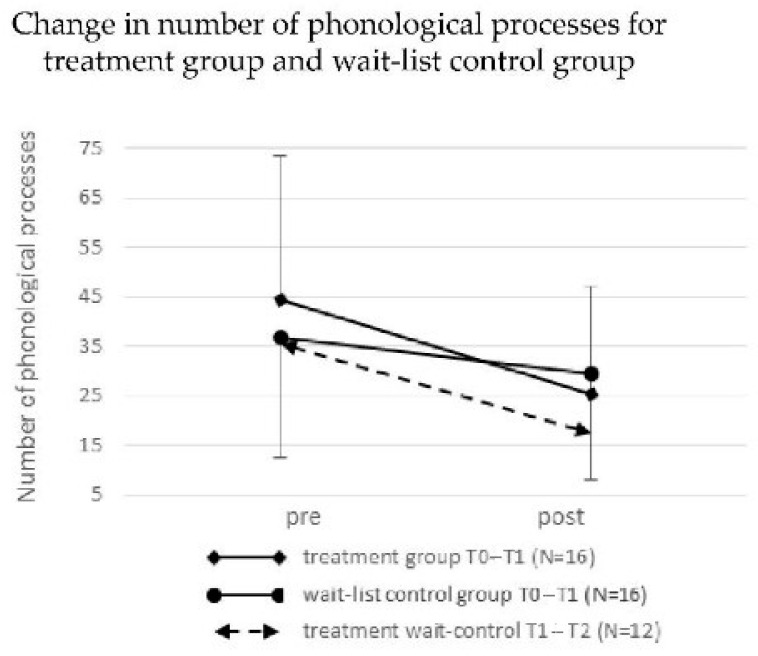
Change in number of phonological processes from To to T1, for the mean of the treatment group and the wait-list control group (solid lines). Change in number of phonological processes from T1 to T2 for the wait-list control group after their treatment phase following the waiting phase is shown as dashed line.

The mean number of phonological processes at T0 was 44.4 for the treatment group and 37.0 for the wait-list control group (Figure 4). This numerical difference, expressed in transformed normalized scores, was not significant (*t*-test, *p* = 0.44). A one-way ANOVA between groups was calculated, with the percentage change in the number of phonological processes from T0 to T1 as the dependent variable and group (treatment vs. wait-list control) as the independent variable. The ANOVA showed a significant main effect for group, F(1,30) = 8.03, *p* = 0.01, and a partial *η^2^* = 0.21. From T1 to T2, the 12 now-treated wait-list control children decreased their number of phonological processes from a mean of 35.6 (*SD* = 21.3, range 7–72) to a mean of 17.6 (*SD* = 14.4, range 2–55). The *t*-test for this decrease, calculated on the basis of normalized distributions, provided significant within-group differences (*t* = 4.47, *df* = 11, *p* = 0.001, *d* = 1.00). Both the original treatment group and the controls treated after the waiting period thus showed similar reductions in the number of phonological processes during their treatment phases (Figure 4). Numerical details are given in Table 3.

### 3.3. Spontaneous Improvements of Misarticulated Phonemes

Not every target phoneme had to be included before reaching the success criterion (occurrence of at least three correct utterances of each target phoneme in the child’s spontaneous speech within a session). Of the 28 children who received therapy, on average, only 34.4% (3.3 out of 9.6) of the target phonemes needed treatment in order to achieve normalization of all target phonemes.

When examining the number of different error phonemes (not the total number of incorrect phonemes) that a child showed at T1, it was noted that children in the treatment group reduced the number of different misarticulated phonemes by 18.8%; in contrast, children in the wait-list control group showed a slight increase of 2.2%.

### 3.4. Reaching Success Criterion

The number of treatment sessions required to achieve the success criterion ranged from 15 to 66, with a mean of 28 (*SD* = 14.6, median = 20). Of the 28 participants, 19 required 15–30 sessions. Seven participants received 38–47 sessions (including a break in treatment after 30 sessions). Two participants completed their treatment after 60–66 sessions (including two treatment breaks).

The treatment time taken to reach the success criterion ranged from 4 to 32 months, with a mean duration of 11.5 months (*SD* = 8.5, median = 7). All 28 children treated reached the treatment success criterion: 21 of them before regular entry into elementary school and the other seven during first grade. A treatment frequency of one session per week was targeted, but because of vacation and illness, a mean weekly treatment frequency of 0.69 was achieved.

### 3.5. Inter-Rater Agreement

In order to assess the reliability of the analysis of the audiotaped utterances on the naming test (PLAKSS), the outcome measures were tallied by an SLP not involved in the treatment and with no knowledge of the children or the time points of testing (T0, T1, and T2). In order to verify interrater reliability, a second independent SLP phonetically transcribed and analyzed 30 randomly selected tapes of the PLAKSS test from the three assessment points and determined the number of incorrectly produced consonants. The scores of both SLPs correlated with *r* = 0.91.

### 3.6. Age, Gender, Parental Educational Level, and Treatment Success

A one-way between-group MANOVA was performed in order to investigate the possible influence of gender, age at T0, and parental educational level on the scores from T0 to T1. The dependent variables used were: (1) gain in PCC and (2) percentage change of the number of phonological processes from T0 to T1. No significant differences (Wilks’ Lambda) were found for gender (F2, 0.758, *p* = 0.48), age at T0 (F2, 2.662, *p* = 0.09), or parental educational level (F2, 0.65, *p* = 0.53).

## 4. Discussion

The results of this randomized controlled trial suggest that the integrated approach applied here may be effective in treating SSD in children. The comparable distributions of age, gender, PCC scores, and the number of phonological processes at T0 indicate successful randomization of study participants. By providing the therapist with a precise description of its components and procedural modifications, PhonoSens allows for replicable, patient-tailored individual treatment pathways, as required by modern evidence-based medicine. The PCC results for words in a naming test improved significantly more for the treatment group than for the control group between T0 and T1, with a large effect size (partial *η^2^* = 0.20). The reduction in the number of phonological processes during treatment was also significantly greater in the treatment group than in the controls, again with a clear effect size (partial *η^2^* = 0.21). Children in the wait-list control group showed the same degree of improvement during the therapy phase subsequent to their waiting period as the children in the treatment group. The latter result can be considered an internal replication of the effectiveness of PhonoSens.

Although spontaneous improvement in articulation can be expected in children with SSD (improvement is usually insufficient to compensate for a delay of more than 6 months and becomes increasingly unlikely after about 5 years of age [15]), the reduction in the number of error phonemes by an average of 20% in the treatment group and by only 0.7% in the control group between time points T0 and T1 suggests that the former provides greater benefit. Five out of sixteen control children did not improve during the waiting period, and two of them deteriorated slightly. In contrast, all children in the treatment group showed progress over the treatment period (T0–T1). The number of misarticulated phonemes over the period from T0 to T1 decreased by 18.8% in the treatment group and increased slightly (2.2%) in the wait-list control group. This shows that not only did the number of mispronounced phonemes decrease but so did the number of different error types, pointing to a treatment effect rather than spontaneous improvement.

Self-monitoring is the highest priority in PhonoSens, and stimulability was, therefore, the most important criterion in the selection of target phonemes. Neither the developmental approach (in which the order of target phonemes is based on phonological development) nor the complexity approach (in which treatment focuses on phonemes and phoneme clusters acquired later in development) was used for sequencing the target phonemes. Nevertheless, only one in three target phonemes needed to be treated. As has been found with the complexity approach [48], significant spontaneous improvement or generalization of untreated phonemes was observed with the PhonoSens treatment in our study.

The use of SSP cards has been shown to be a successful means of identifying and distinguishing error and target phonemes, similar to the use of letters in Rvachew’s study [22]. Letters, however, run the risk of phoneme–grapheme incongruence. This risk is not inherent in SSP cards and so may be beneficial for later reading–writing acquisition. A further advantage of using SSP cards is that children might name and identify the associated phonemes more easily when using the symbolized non-speech sounds on the SSP cards than when using random graphic symbols (letters).

An SSD therapy study by Goorhuis-Brouwer and Knijff [64] demonstrated adequate speech and language skills in only a small number of children after 12 months of treatment, half of whom needed further treatment. In comparison, in the study presented here, more than two-thirds of children achieved the success criterion in less than one year and almost half within six months. Less than one-third of children needed further treatment after 12 months. These findings question the assumption of Goorhuis-Brouwer and Knijff [64] that watchful waiting might be indicated.

The two children who had the longest treatment times in our study required intensive and lengthy training at each treatment step; in particular, the development of their auditory self-monitoring skills was laborious. In contrast, those children who successfully completed their therapy after 15 sessions were able to produce the target phonemes correctly at an earlier stage of treatment and already showed generalization of the target phoneme into spontaneous speech at the phase of *categorical phonological perception*.

The results of this study suggest that phonology as linguistic performance and phonetics as motor performance of speech sound development and treatment should not be considered separately. Moreover, neuroimaging studies and derived models of the neurobiology of language indicate that extensive temporal, frontoparietal, cerebellar, and subcortical brain networks are highly interconnected in speech perception and production, working together in parallel or sequentially in a finely tuned temporal sequence (e.g., [40,65,66,67]), a finding that is also reflected in the IPMSP model of Terband et al. [32]. We assume that the integrative character of PhonoSens, which combines auditory and somatosensory self-perception with speech planning and speech motor elements, supported by TTOS, could train large parts of these networks. Electrophysiological and further functional and structural neuroimaging studies may shed light on the connectivity of these networks.

The RCT by Almost and Rosenbaum [20] is most closely comparable to our RCT. In their study, 30 children (15 per group) were assessed in a wait-contrast design. The direct treatment group received a 4-month intervention, beginning with the minimal pair approach of Hodson and Paden [68], followed by classic articulation therapy. The treatment period was followed by a 4-month waiting period. The control group received no therapy for the first 4 months and then began a 4-month therapy period. The results showed significant improvement during the treatment period for the direct treatment group over with the wait-list control group. However, the number of sessions varied considerably between treatment and control groups (14 and 29 sessions, respectively). In the study reported here, all children received 15 therapy sessions, but the total duration of treatment varied (4–9 months). Both studies indicate that a combination of phonological and phonetic aspects leads to significant improvement. In contrast to the approach of Almost and Rosenbaum [20], our study augments phonological and phonetic aspects with internal and external feedback training. Our long-term evaluation confirms the effectiveness of the PhonoSens treatment approach. The use of four therapists in our study compared to one in the Almost and Rosenbaum study [20] supports its generalizability.

Therapy methods such as minimal pairs [68] have strongly influenced the therapeutic landscape for SSD treatment within English-speaking nations. In German-speaking countries, methods such as psycholinguistically oriented phonology therapy (POPT) have had a similarly important influence on the treatment of SSD [15]. Although there are some similarities between POPT and PhonoSens, such as the use of reference cards of non-speech sounds symbolizing phonemes, there are also significant differences. In POPT, the child is told at the beginning of therapy that they need to change something about their speech. PhonoSens, however, stimulates change by intensively reinforcing self-monitoring skills without directly addressing a child’s speech sound deficits. POPT divides SSD into different subgroups and uses different approaches for each type (as does Dodd [33]). PhonoSens does not subdivide but offers a flexible treatment pathway depending on the progress of treatment for each target phoneme and the time point of the onset of generalization of the target phoneme in spontaneous speech. A further significant difference is that POPT targets complete phonological processes and treats all affected phonemes simultaneously. In PhonoSens, phonemes are addressed one at a time, with a maximum of two phonemes being treated simultaneously at a single stage of therapy, and this method still achieves a corresponding change in the child’s phonological system, as evidenced by the spontaneous improvement of an average of two-thirds of the target phonemes defined at the beginning of therapy. POPT and PhonoSens both use hierarchical treatment steps via isolated phonemes and syllables to the word level, as do many other methods. POPT adds levels with different non-word categories between the syllable and the word level, but PhonoSens does not. Both methods employ a thermic stimulus: in POPT, this is used to improve the child’s intraoral orientation for velar and alveolar sounds; in PhonoSens, the thermal stimulus is used to support motor learning and a hypothesized associated increase in cortical activation. Finally, the targeted frequency of the therapy sessions also differs, with POPT taking place twice a week and our study offering PhonoSens once a week, which is the usual frequency in Germany [63].

The present study was originally intended to enroll a population-based sample, with the intention of including all children from the indicated age group in the five participating kindergartens. Unfortunately, a number of parents did not agree to participate. We assume that parents who suspected a deficit in their child’s language acquisition were more likely to agree to the screening tests than those who did not. Moreover, children of parents who did not respond to the invitation letter may already have been in treatment. Although the proportion of children with SSD found in this study (17.7%) is within the range of reported prevalence values (from 2.3% to 24.6% [5]), it is still relatively high, possibly justified by selection bias due to the problems outlined above.

Surprisingly, our study found that more girls than boys were diagnosed with SSD (boys to girls: 1:1.3). Speech–language disorders typically affect more boys than girls, and this is also true for SSD. Out of 1100 referrals to a pediatric speech and language service, Broomfield and Dodd [6] found that a boy-to-girl ratio for developmental language disorders of approx. 3:1 existed and, for SSD, slightly less than 2:1. One would expect this gender ratio to be reflected in the utilization of therapy; however, according to a recent Dutch study, girls with speech and language disorders were admitted to speech and language centers on average 5 months (in some regions even 10 months) later than boys with comparable deficits [69]. The unusual gender ratio in our study could, therefore, be due to recruitment in kindergartens, with a higher likelihood of contact with girls who had not yet received therapy than with boys.

The treatment approach presented here may be similarly effective for languages other than German because the categorical perception of phonemes and auditory self-monitoring are required for speech sound acquisition in any language. One cross-linguistic review [70] showed that children acquire most consonants of their native language by around the age of 5.0 years. Some basic developmental principles seem to be similar in different languages, such as the earlier acquisition of plosives than fricatives. It, therefore, seems reasonable to also investigate the effectiveness of the PhonoSens approach for languages other than German.

### Limitations

The mean PCC scores and the number of phonological processes found at time point T0 differed numerically between the treatment and the wait-list control group, but not significantly. This difference might impact the comparability of both groups. We, therefore, determined the degree of improvement shown by the children in the wait-list control group during their treatment phase. Both measures of treatment effectiveness were of comparable magnitude to those of the children in the treatment group. This concern about the comparability of both groups seems, therefore, to be unfounded.

Because of holidays and illnesses, the individual time spans recorded between the T0 and T1 treatment or waiting times varied considerably, from four to nine months, with a mean duration of six months found in both groups. In daily practice, intervention intensity cannot be controlled as well as in an efficacy study. Intervention studies can be classified on a continuum between experimental (efficacy studies) and pragmatic (effectiveness studies) [71]. Efficacy studies investigate the effect of an intervention under strictly controlled experimental conditions and have high internal validity. Effectiveness studies are more pragmatic and reflect everyday clinical practice, in which conditions vary. Effectiveness studies have lower internal validity but higher external validity, meaning that results are more generalizable to the entire population. These two types of studies complement each other by balancing internal and external validity differently; thus, both provide valuable contributions to the understanding of an intervention and its application in clinical practice. The criteria for the classification of intervention studies suggest that the study presented here would likely be assigned to the pragmatic, effectiveness study type [72]. Specifically, the research question (intervention effect in everyday practice) and the setting (clinical practice) clearly belong to the pragmatic study design, and, as a consequence of these design elements, the time spans between tests varied considerably. However, other items met criteria that were halfway between experimental and pragmatic. For example, there were specific inclusion and exclusion criteria for subjects (e.g., no comorbidities, no other therapies, SSD only), but baseline PCC scores varied between groups. As a result, internal validity was weakened by the heterogeneity of the groups and the different time spans over which outcomes were measured, but external validity was strengthened by the greater generalizability of the outcome.

Even though the sample size in our study is relatively large in comparison with other relevant studies, it is still small. Further investigation with larger sample size is therefore desirable.

The use of TTOS is certainly the least evidence-based component of PhonoSens treatment. Although it can be assumed, on the basis of the above-mentioned studies on dysphagia therapy and on the effect of mechanical facial distortions on speech sound perception, that TTOS could also be effective in SSD therapy, this requires proof and should be investigated in a follow-up study focusing on the contribution of individual therapy constituents.

## 5. Conclusions

The results of the study presented here suggest that integrated treatment methods, such as the PhonoSens method applied here, are effective in treating SSD in children. This is the first such finding within the community of German professionals engaged in the treatment of developmental speech and language disorders. The inclusion of different pathways within PhonoSens and its thoroughly described approach allows for child-tailored adaptation of the course of treatment. This study underlines the need to consider speech sound development and treatment as a complex of phonological and phonetic aspects and to reinforce the role of internal and external feedback mechanisms in SSD treatment as conceptualized in the Integrated Psycholinguistic Model of Speech Processing.

This effectiveness study was conducted under daily practice conditions with an average treatment frequency of 0.69 per week. The participants required a mean of 28 sessions and successfully completed their treatment within a mean of 11.5 months. We found that only one-third of target phonemes needed treatment before the success criterion (the start of generalization of all target phonemes into spontaneous speech) was reached. The spontaneous improvement of the untreated phonemes was found only in the treatment phases, not in the waiting phase.

The most valuable next steps in this research area would be to focus on comparing different treatment settings, such as individual vs. group therapy [73], extensive vs. intensive therapy, traditional vs. tablet-based treatment [74]. Since the general concept of this treatment seems to transfer well to different languages, further studies could beneficially investigate the effectiveness of PhonoSens in languages other than German.

## Figures and Tables

**Table 1:** Processing and monitoring levels involved in the speech production chain according to the IPMSP model by Terband et al. [23] and the corresponding steps of the PhonoSens treatment concept.

Realization of the IPMSP Model in PhonoSens	
Processing/Monitoring Levels of IPMSP Model	Realization in the PhonoSens Concept
Processes	PhonoSens steps
*Phonological encoding:*	*Step 1: Categorical phonological perception*
Selection and sequencing of linguistic units that target the formation of phonological units	Training of categorical phonological perception for target phonemes, error phonemes, and acoustically similar phonemes
*Motor planning:*	*Step 2: Phoneme acquirement*
Articulatory movement targets are selected	Stepwise initiation of the target phoneme in an increasingly complex coarticulatory context up to the sentence level
*Motor programming:*	*Step 3: Articulatory fine-tuning I*
Muscle-specific motor planning	Phoneme production training in additional words and sentences
*Motor execution:*	*Step 4: Articulatory fine-tuning II*
Neural signals are transmitted to peripheral systems and converted into coordinated muscle activity	Phoneme production training for words that are repeated three times quickly in one breath
Monitoring	PhonoSens steps
*Internal self-monitoring:*	*Step 5: Enhanced auditory self-monitoring*
Incorrectly planned phonemes are detected	This step focuses on the planned decision for the correct use (selection and localization) of the target phoneme in words
*External auditory self-monitoring:*	*Step 6: Assisted generalization*
Error correction and instant articulatory adaptation	This step focuses on improvement of the child’s self-correction during spontaneous speech. Steps 5 and 6 both address internal, external auditory, and somatosensory self-monitoring processes
*External somatosensory self-monitoring:*	*TTOS: Thermal Tactile Oral Stimulation*
Information regarding the current state of the articulators and necessary error correction	Each treatment begins with a short thermal tactile oral stimulation (TTOS) using an ice-stick. The TTOS is believed to facilitate speech motor learning by temporarily increasing cortical somatosensory activity.

**Table 2:** The columns “incorrect consonants” list the mispronounced consonants; consonants mispronounced at least twice are written in bold; consonants treated from T0 to T1 are underlined; the columns “Conson” show the total number of mispronounced consonants and consonant clusters.

Mispronounced Consonants per Participant						
	Age at T0		Assessment T0		Assessment T1	
Child	(y;m)	Gender	Incorrect Consonants	Conson	Incorrect Consonants	Conson
Treatment group						
1	5;06	f	m p f l n t j x g k s ʃ	38	m n t k s	18
5	4;10	f	m b p v l n t ç *g* ŋ k ʃ	40	m b l n g ʃ	11
7	3;07	f	*m* b f v l n t j *ç* ʁ *g* ŋ k z s ʃ	100	m b f v l n d t ç ʁ g k s ʃ	52
8	4;09	m	f l n t ç ʁ g k ʃ	13	k ʃ	2
10	4;04	f	m b p n t g k s ʃ	39	m b n t ʁ g k z s ʃ	31
13	5;05	m	f l t ç g ŋ k ʃ	47	f v l t j ç k z s ʃ	41
14	4;05	m	m l n t ç g k ʃ	32	l n t ç x g k s ʃ	18
16	5;02	f	m n t ç g ŋ k s ʃ	59	n t ç	4
18	3;07	f	f v l n d t ç g ŋ k s ʃ	64	l n d t ç g k ʃ	42
19	5;01	m	n t g k ʃ	10	f l n ç g k ʃ	8
20	4;10	f	l t ç ʁ g s ʃ	18	v n ç g ʃ	7
23	4;05	m	p l n t ʁ g ŋ k ʃ	56	f v n t ʁ g ŋ k s ʃ	54
26	5;00	f	b l n t ç ʁ g ŋ k s ʃ	25	m b l n t h ç g k ʃ	17
27	5;00	f	l n d t ç g ŋ k s ʃ	32	m p v n t ç g k s ʃ	16
28	4;02	m	p l n t ç g k ʃ	16	n t g k	8
29	4;02	m	p f v l n t j ç ʁ g ŋ k s ʃ	91	m b v n d t ç ʁ g ŋ k s ʃ	64
Wait-list control group						
2	4;07	f	b p f v n t ç ʁ g k z ʃ	41	m b p f v l n t j ç g z s ʃ	44
3	4;05	f	b l n d t j ç g k ʃ	40	m f n d t ç x ʁ g z ʃ	24
4	4;01	f	m p f v l n t j ç x ʁ g k s ʃ	77	v l t ç ʁ g ŋ k s ʃ	58
6	4;06	f	f l n t ç g ŋ k ʃ	24	m b p f l n t g ŋ k s ʃ	20
9	4;09	f	v t g k ʃ	8	m t ŋ s ʃ	6
11	5;01	f	f l d t ç g k s ʃ	16	n d t k	9
12	4;01	f	m v l n d t ç ʁ g k ʃ	44	m p v l n t j ç g k s ʃ	44
15	4;06	m	m b p v l n t ç g k s ʃ	27	m f l n d t ʁ g k s ʃ	19
17	4;10	f	t ç k ʃ	7	n t ç g ʃ	7
21	4;11	f	f n t g s ʃ	15	m l n t ʁ g k ʃ	16
22	3;09	m	m l n t ç ʁ g k s ʃ	45	b v l n t ç ʁ z ʃ	31
24	3;09	m	b p v l n t ç g k s ʃ	76	p v l n t ç g ŋ k ʃ	56
25	4;02	m	l n d t ʁ g ŋ k s ʃ	46	m b p n d t j g ŋ k s ʃ	45
30	4;06	m	l t ʃ	31	n ʃ	2
31	5;00	m	b g ʃ	22	n t ŋ k ʃ	26
32	4;07	m	f l t g ŋ k	43	m t g ŋ k s ʃ	42

**Table 3:** Number of phonological processes per participant. Their change from T0 to T1 is given in column # pp diff. T0–T1.

Number of Phonological Processes per Participant					
	Age at T0		Assessment T0	Assessment T1	
Child	(y;m)	Gender	# Phonological Processes	# Phonological Processes	# pp diff. T0-T1
Treatment group					
1	5;06	f	34	18	−16
5	4;10	f	42	10	−32
7	3;07	f	112	59	−53
8	4;09	m	15	2	−13
10	4;04	f	39	31	−8
13	5;05	m	50	37	−13
14	4;05	m	29	19	−10
16	5;02	f	57	3	−54
18	3;07	f	70	43	−27
19	5;01	m	10	9	−1
20	4;10	f	16	6	−10
23	4;05	m	67	59	−8
26	5;00	f	27	17	−10
27	5;00	f	35	17	−18
28	4;02	m	16	7	−9
29	4;02	m	92	69	−23
Wait-list control group					
2	4;07	f	41	43	2
3	4;05	f	40	21	−19
4	4;01	f	88	72	−16
6	4;06	f	25	21	−4
9	4;09	f	7	6	−1
11	5;01	f	15	8	−7
12	4;01	f	51	39	−12
15	4;06	m	23	17	-6
17	4;10	f	7	7	0
21	4;11	f	15	17	2
22	3;09	m	49	34	−15
24	3;09	m	86	70	−16
25	4;02	m	49	44	−5
30	4;06	m	32	2	−30
31	5;00	m	21	26	5
32	4;07	m	43	46	3

## Data Availability

All data used to support the finding of this study are available from the corresponding author upon request. The data are not publicly available because they concern the privacy of children.

## References

[1] World Health Organization ICD-11 International Classification of Diseases 11th Revision. https://icd.who.int/en/.

[2] Felsenfeld S., Broen P.A., McGue M. (1994). A 28-year follow-up of adults with a history of moderate phonological disorder: Educational and occupational results. J. Speech Hear. Res..

[3] McCormack J., McLeod S., McAllister L., Harrison L.J. (2009). A systematic review of the association between childhood speech impairment and participation across the lifespan. Int. J. Speech Lang. Pathol..

[4] Von Suchodoletz W. (2009). Wie wirksam ist Sprachtherapie? [How effective is speech therapy?]. Kindh. Und Entwickl..

[5] Wren Y., Miller L.L., Peters T.J., Emond A., Roulstone S. (2016). Prevalence and predictors of persistent speech sound disorder at eight years old: Findings from a population cohort study. J. Speech Lang. Hear. Res..

[6] Broomfield J., Dodd B. (2004). Children with speech and language disability: Caseload characteristics. Int. J. Lang. Commun. Disord..

[7] Campbell T.F., Dollaghan C.A., Rockette H.E., Paradise J.L., Feldman H.M., Shriberg L.D., Sabo D.L., Kurs-Lasky M. (2003). Risk factors for speech delay of unknown origin in 3-year-old children. Child Dev..

[8] Fox-Boyer A.V., Glück C.W., Elsing C.E., Siegmüller J., Fox-Boyer A. (2014). Erwerb von Phonologie, Lexikon und Grammatik bei Kindern im Alter von 3; 0–5; 0 Jahren [Acquisition of phonology, lexicon, and grammar in 3- to 5-year-old children]. Handbuch Spracherwerb und Sprachentwicklungsstörungen. Kindergartenphase [Handbook of Language Acquisition and Language Development Disorders. Kindergarten Phase].

[9] Shriberg L.D., Kwiatkowski J. (1990). Self-monitoring and generalization in preschool speech-delayed children. Lang. Speech Hear. Serv. Sch..

[10] Van Borsel J. (2019). Basisbegrippen Logopedie, Deel 2: Communicatiestoornissen [Basic Concepts of Speech Therapy, Part 2: Communication Disorders].

[11] Weggemans M. (2005). Jaarverslag Logopedie 2004–2005 [Annual Report Speech Therapy 2004–2005].

[12] Waltersbacher A., WIdO—Institut der AOK Heilmittelbericht 2019 Ergotherapie, Sprachtherapie Physiotherapie, Podologie. [Cure Report: Occupational Therapy Speech Therapy Physiotherapy Podiatry]; Berlin, Germany, 2019. https://www.wido.de/fileadmin/Dateien/Dokumente/Publikationen_Produkte/Buchreihen/Heilmittelbericht/wido_hei_hmb_2019.pdf.

[13] American Speech-Language-Hearing Association (n.d.) Speech sound disorders: Articulation and phonology. (Practice Portal). www.asha.org/Practice-Portal/Clinical-Topics/Articulation-and-Phonology.

[14] Gironda F., Fabus R., Stein C., Fabus R. (2011). Assessment of articulation and phonological disorders. A Guide to Clinical Assessment and Professional Report Writing in Speech-Language Pathology.

[15] Fox-Boyer A.V. (2016). Kindliche Aussprachestörungen [Speech Sound Disorders in Children].

[16] Wertzner H., Amaro L., Teramoto S.S. (2005). Severity of phonological disorders: Perceptual judgement and percentage of correct consonants. Pró Fono Rev. De Atualizaçã Científica.

[17] Law J., Garrett Z., Nye C. (2004). The efficacy of treatment for children with developmental speech and language delay disorder: A meta-analysis. J. Speech Lang. Hear. Res..

[18] Nelson H.D., Nygren P., Walker M., and Panoscha R. (2006). Screening for speech and language delay in preschool children: Systematic evidence review for the US Preventive Services Task Force. Pediatrics.

[19] Glogowska M., Roulstone S., Enderby P., Peters T.J. (2000). Randomized controlled trial of community based speech and language therapy in preschool children. Br. Med. J..

[20] Almost D., Rosenbaum P. (1998). Effectiveness of speech intervention for phonological disorders: A randomized controlled trial. Dev. Med. Child Neurol..

[21] Wren Y., Harding S., Goldbart J., Roulstone S. (2018). A systematic review and classification of interventions for speech-sound disorder in preschool children. Int. J. Commun. Disord..

[22] Rvachew S., Nowak M., Cloutier G. (2004). Effect of phonemic perception training on the speech production and phonological awareness skill of children with expressive phonological delay. Am. J. Speech Lang. Pathol..

[23] Weiner F.F. (1981). Treatment of phonological disability using the method of meaningful minimal contrast: Two case studies. J. Speech Hear. Disord..

[24] Baker E., McLeod S. (2011). Evidence-Based practice for children with speech sound disorders: Part 1 Narrative Review. Lang. Speech Hear. Serv. Sch..

[25] Yoder P., Camarata S., Gardner E. (2005). Treatment effects on speech intelligibility and length of utterance in children with specific language and intelligibility impairments. J. Early Interv..

[26] Howell J., Dean E.C. (2004). Treating Phonological Disorders in Children: Metaphon Theory to Practice.

[27] Dodd B., Holm A., Crosbie S., McIntosh B., Williams A.-L., McLeod S., McCauly R. (2010). Core vocabulary intervention. Interventions for Speech Sound Disorders in Children.

[28] Crosbie S., Holm A., Dodd B. (2005). Intervention for children with severe speech disorder: A comparison of two approaches. Int. J. Lang. Comm. Dis..

[29] Pamplona M.C., Ysunza A., Espinosa J. (1999). A comparative trial of two modalities of speech intervention for compensatory articulation in cleft palate children, phonological approach versus articulatory approach. Int. J. Pediatric Otorhinolaryngol..

[30] Lousada M., Jesus L., Capelas S., Margaça C., Tomé D., Valente R., Hall A., Joffe V. (2013). Phonological and articulation treatment approaches in Portuguese children with speech and language impairments: A randomized controlled intervention study. Int. J. Lang. Commun. Disord. R. Coll. Speech Lang. Ther..

[31] Dodd B., Bradford A. (2000). A comparison of three therapy methods for children with different types of developmental phonological disorder. Int. J. Lang. Commun. Disord..

[32] Terband H., Maassen B., Maas E.A. (2019). Psycholinguistic framework for diagnosis and treatment planning of developmental speech disorders. Folia Phoniatr. Logop..

[33] Dodd B. (2014). Differential diagnosis of pediatric speech sound disorder. Curr. Dev. Disord. Rep..

[34] Dworkin J.P., Marunick M.T., Krouse J.H. (2004). Velopharyngeal dysfunction: Speech characteristics, variable etiologies, evaluation techniques, and differential treatments. Lang. Speech Hear. Serv. Sch..

[35] Kummer A.W., Marty-Grames L., Jones D.L., Kurnell M.P., Ruscello D. (2006). Response to “velopharyngeal dysfunction: Speech characteristics, variable etiologies, evaluation techniques, and differential treatments” by Dworkin, Marunick, and Krouse, October 2004. Lang. Speech Hear. Serv. Sch..

[36] Namasivayam A.K., Coleman D., O’Dwyer A., van Lieshout P. (2020). Speech sound disorders in children: An articulatory phonology perspective. Front. Psychol..

[37] Van Lieshout P., Maassen B., Kent R., Herman P., van Lieshout P., Woulter H. (2004). Dynamical systems theory and its application in speech. Speech Motor Control in Normal and Disordered Speech.

[38] Namasivayam A.K., van Lieshout P. (2011). Speech motor skill and stuttering. J. Motor. Behav..

[39] Furlong L.M., Morris M.E., Serry T.A., Erickson S. (2021). Treating childhood speech sound disorders: Current approaches to management by Australian speech-language pathologists. Lang. Speech Hear. Serv. Sch..

[40] Guenther F.H. (1994). A neural network model of speech acquisition and motor equivalent speech production. Biol. Cybern..

[41] Guenther F., Perkell J., Maassen B., Kent R., Peters H.F.M., van Lieshout P., Hulstijn W. (2004). A new model of speech production and its application to studies of the role of auditory feedback in speech. Speech Motor Control in Normal and Disordered Speech.

[42] Tourville J.A., Guenther F.H. (2011). The DIVA model: A neural theory of speech acquisition and production. Lang. Cogn. Process..

[43] Levelt W.J., Roelofs A., Meyer A.S. (1999). A theory of lexical access in speech production. Behav. Brain Sci..

[44] Kühnert B., Nolan F., Hardcastle W.J., Hewlett N. (1999). The origin of coarticulation. Coarticulation. Theory, Data and Techniques.

[45] Özdemir R., Roelofs A., Levelt W.J. (2007). Perceptual uniqueness point effects in monitoring internal speech. Cognition.

[46] Koegel L.K., Koegel R.L., Costello Ingham J. (1986). Programming rapid generalization of correct articulation through self-monitoring procedures. J. Speech Hear. Disord..

[47] Wolfe V., Presley C., Mesaris J. (2003). The importance of sound identification training in phonological intervention. Am. J. Speech-Lang. Pathol..

[48] Gierut J.A., Champion A.H. (2001). Syllable onsets II: Three-element clusters in phonological treatment. J. Speech Lang. Hear. Res..

[49] Storkel H.L. (2018). The complexity approach to phonological treatment: How to select target phonemes. Lang. Speech Hear. Serv. Sch..

[50] Cochrane A.L. (1972). Effectiveness and Efficiency: Random Reflection on Health Services.

[51] Haynes B. (1999). Can it work? Does it work? Is it worth it?. Br. Med. J..

[52] Baker E., Williams A.L., McLeod S., McCauley R. (2018). Elements of phonological interventions for children with speech sound disorders: The development of a taxonomy. Am. J. Speech Lang. Pathol..

[53] Hesketh A., Adams C., Nightingale C., Hall R. (2000). Phonological awareness therapy and articulatory training approaches for children with phonological disorders: A comparative outcome study. Int. J. Lang. Commun. Disord..

[54] Fox A.V. (2009). PLAKSS—Psycholinguistische Analyse Kindlicher Sprechstörungen [Psycholinguistic Analysis of Speech Sound Disorders in Childhood].

[55] Möttönen R., Watkins K.E. (2009). Motor representations of articulators contribute to categorical perception of speech sounds. J. Neurosci..

[56] Rvachew S., Jamieson D.G. (1989). Perception of voiceless fricatives by children with a functional articulation disorder. J. Speech Hear. Disord..

[57] Teismann I.K., Steinsträter O., Warnecke T., Suntrup S., Ringelstein E.B., Pantev C., Dziewas R. (2009). Tactile thermal oral stimulation increases the cortical representation of swallowing. BMC Neurosci..

[58] Ito T., Tiede M., Ostry D.J. (2009). Somatosensory function in speech perception. Proc. Natl. Acad. Sci. USA.

[59] Nasir S.M., Ostry D.J. (2009). Auditory plasticity and speech motor learning. Proc. Natl. Acad. Sci. USA.

[60] Sugden E., Baker E., Munro N., Williams A.L., Trivette C.M. (2018). Service delivery and intervention intensity for phonological-based speech sound disorders. Int. J. Lang. Commun. Disord..

[61] Williams A.L. (2012). Intensity in phonological intervention: Is there a prescribed amount?. Int. J. Speech Lang. Pathol..

[62] Neumann K., Holler-Zittlau I., van Minnen S., Sick U., Zaretsky Y., Euler H.A. (2011). Katzengoldstandards in der Sprachstandserfassung: Sensitivität und Spezifität des Kindersprachscreenings (KiSS). [Fool’s gold standards in language screening. Sensitivity and specificity of the Hessian child language screening test (Kindersprachscreening, KiSS)]. HNO.

[63] Ritterfeld U., Rindermann H. (2004). Mütterliche Einstellung zur sprachtherapeutischen Behandlung ihrer Kinder [Maternal attitudes toward speech therapy treatment of their children]. Z. Für Klin. Psychol. Und Psychother..

[64] Goorhuis-Brouwer S.M., Knijff W.A. (2003). Language disorders in children: When is speech therapy recommend?. Int. J. Pediatric Otorhinolaryngol..

[65] Hickok G. (2009). The functional neuroanatomy of language. Phys. Life Rev..

[66] Hickok G., Poeppel D. (2004). Dorsal and ventral streams: A framework for understanding aspects of the functional anatomy of language. Cognition.

[67] Kotz S.A., Schwartze M. (2010). Cortical speech processing unplugged: A timely subcortico-cortical framework. Trends Cogn. Sci..

[68] Hodson B., Paden E. (1991). Targeting Intelligible Speech: A Phonological Approach to Remediation.

[69] Wiefferink K., van Beugen C., Wegener Sleeswijk C., Gerrits E. (2020). Children with language delay referred to Dutch speech and hearing centres: Caseload characteristics. Int. J. Lang. Commun. Disord..

[70] McLeod S., Crowe K. (2018). Children’s consonant acquisition in 27 languages: A cross-linguistic Review. Am. J. Speech Lang. Pathol..

[71] Gartlehner G., Hansen R.A., Nissman D., Lohr K.N., Carey T.S. (2006). A simple and valid tool distinguished efficacy from effectiveness studies. J. Clin. Epidemiol..

[72] Singal A.G., Higgins P.D.R., Waljee A.K. (2014). A Primer on Effectiveness and Efficacy Trials. Clin. Transl. Gastroenterol..

[73] Ottow-Henning E., Keij B. (2020). Does group intervention make a difference for the speech sound development of Dutch pre-school children with Developmental Language Disorder?. Int. J. Speech Lang. Pathol..

[74] Jesus L.M.T., Martinez J., Santos J., Hall A., Joffe V. (2019). Comparing traditional and tablet-based intervention for children with speech sound disorders: A randomized controlled trial. J. Speech Lang. Hear. Res..

